# MicroRNA-34a (miR-34a) Mediates Retinal Endothelial Cell Premature Senescence through Mitochondrial Dysfunction and Loss of Antioxidant Activities

**DOI:** 10.3390/antiox8090328

**Published:** 2019-08-22

**Authors:** Menaka C. Thounaojam, Ravirajsinh N. Jadeja, Marie Warren, Folami L. Powell, Raghavan Raju, Diana Gutsaeva, Sandeep Khurana, Pamela M. Martin, Manuela Bartoli

**Affiliations:** 1 Department of Ophthalmology, Medical College of Georgia, Augusta University, Augusta, GA 30912, USA; 2 Department of Biochemistry and Molecular Biology, Medical College of Georgia, Augusta University, Augusta, GA 30912, USA; 3 Department of Pharmacology and Toxicology, Medical College of Georgia, Augusta University, Augusta, GA 30912, USA; 4 Division of Gastroenterology, Hepatology and Nutrition and Weight Management, Geisinger Medical Center, Danville, PA 17822, USA

**Keywords:** diabetic retinopathy, mitochondrial dysfunction, miR-34a, vascular senescence

## Abstract

Stress-associated premature senescence (SAPS) is involved in retinal microvascular injury and diabetic retinopathy. We have investigated the role and mode of action of miR-34a in retinal endothelial cells senescence in response to glucidic stress. Human retinal microvascular endothelial cells (HuREC) were exposed to glucidic stress (high glucose (HG) = 25 mM d-glucose) and compared to cells exposed to normal glucose (NG = 5 mM) or the osmotic control l-glucose (LG = 25 mM). HG stimulation of HuREC increased the expression of miR-34a and induced cellular senescence. HG also increased the expression of p16ink4a and p21waf1, while decreasing the histone deacetylase SIRT1. These effects were associated with diminished mitochondrial function and loss of mitochondrial biogenesis factors (i.e., PGC-1α, NRF1, and TFAM). Transfection of the cells with miR-34a inhibitor (IB) halted HG-induced mitochondrial dysfunction and up-regulation of senescence-associated markers, whereas miR-34a mimic promoted cellular senescence and mitochondrial dysfunction. Moreover, HG lowered levels of the mitochondrial antioxidants TrxR2 and SOD2, an effect blunted by miR-34a IB, and promoted by miR-34a mimic. 3’-UTR (3’-untranslated region) reporter assay of both genes validated TrxR2 as a direct target of miR-34a, but not SOD2. Our results show that miR-34a is a key player of HG-induced SAPS in retinal endothelial cells via multiple pathways involved in mitochondrial function and biogenesis.

## 1. Introduction

Diabetic retinopathy (DR) is a potentially blinding complication characterized by progressive retinal neurovascular injury and consequent visual impairment [1,2]. The molecular events involved in DR pathogenesis are still under investigation and their understanding is necessary for the development of new diagnostic and therapeutic tools. 

Recent studies by our group and others have shown that hyperglycemia accelerates vascular senescence through a process known as stress-associated premature senescence (SAPS) [3,4,5]. This pathogenic mechanism appears to be associated with increased oxidative stress and epigenetic silencing of NAD^+^-dependent histone deacetylase sirtuin-1 (silent mating type information regulation 2 homolog) (SIRT1) [6,7,8]. As a consequence, endothelial cells acquire senescence-like features, ultimately leading to expression of inflammatory cytokines and increased apoptosis [7,8,9]. 

Clinical and experimental evidence implicates epigenetic mechanisms, such as differential expression of microRNAs (miRs), in the pathogenic processes involved in diabetes-induced retinal neurovascular injury [10,11].

MicroRNA-34a-5p (miR-34a) has been shown to be a translational suppressor of SIRT1 [12,13] and its function has been implicated in vascular senescence [14,15], oxidative stress [16,17], and apoptosis [12,18]. Altered expression of miR-34a is evident in several human pathologies, including cancer [18,19] and cardiovascular disease [20,21]. Both, experimental and human diabetes are associated with increased expression of miR-34a, which in turn is linked to hyperglycemia-induced vascular dysfunction [22,23,24]. Previously we have showed that miR-34a is upregulated in the diabetic retina [3] and this effect is associated with increased levels of senescence markers and loss of SIRT1 expression and activity [3]. 

Increased oxidative/nitrative stress plays a causal role in retinal vascular dysfunction and hyperglycemia-induced premature senescence [3,25,26]. The contribution of miR-34a to this process is poorly understood and could go beyond its effects as translational inhibitor of SIRT1. 

MiR-34a has been shown to contribute to oxidative stress by altering mitochondrial function [27,28,29]. Whether or not this mechanism is involved in retinal endothelial cell senescence in the diabetic milieu has not been previously investigated. Interestingly, the mitochondrial endogenous antioxidant thioredoxin reductase 2 (TrxR2) has been shown to be a potential target of miR-34a [30], thus suggesting that miR-34a-mediated loss of antioxidants could also contribute to retinal endothelial cells mitochondrial dysfunction leading to premature senescence. 

Based on these collective observations, here we have investigated the specific contribution and mechanism of action of miR-34a in promoting mitochondrial dysfunction and retinal microvascular endothelial cells senescence.

## 2. Materials and Methods 

### 2.1. Cells and Treatment

Human Retinal Endothelial cells (HuREC) were purchased from Cell Systems Corporation (Kirkland, WA, USA). The cells were maintained in CSC complete medium with normal glucose formulation (Cell System Corporation) at 37 °C in a humidified atmosphere of 5% CO_2_ in air as suggested by the vendor. HuREC were used between passages 3 to 7 and cultured in tissue culture flasks pre-coated with attachment factor (Cell Systems Corporation). Ten to 12 h before the experiments the cells were switched to serum-free CSC medium (Cell System Corporation). 

To mimic the effects of the hyperglycemia (glucidic stress), HuREC were cultured for 48 h in serum-free medium containing 25 mM D-glucose (high glucose, HG). Control cells were cultured in serum-free and normal glucose medium (5.5 mM d-glucose, NG) or with the addition of l-glucose (5.5 mM d-glucose + 19.5 mM l-glucose, LG) as osmotic control.

### 2.2. Micro RNA Detection

MiRNAs were isolated using a miRCURY LNA™ Universal RT (Qiagen, Germantown, MD, USA), and cDNA was prepared using a universal cDNA synthesis kit (Qiagen, Germantown, MD, USA) as per manufacturer’s instructions. Quantitative polymerase chain reaction (qPCR) was performed using specific has-miR-34a primer, 5’UGGCAGUGUCUUAGCUGGUUGU 3’ (Qiagen, Germantown, MD, USA), and the ExiLENT SYBR^®^ Green PCR Master Mix (Qiagen, Germantown, MD, USA). The conditions used for qPCR were as it follows: 95 °C for 10 min (1 cycle), 95 °C for 10 s, and 60 °C for 1 min (40 cycles). The thermal cycler StepOne™ Real-Time PCR System (Applied Biosystems, Foster City, CA, USA) was used for qPCR, and the data were analyzed using StepOne™ Plus software (Applied Biosystems, Waltham, MA, USA). Relative miRNA abundance was determined by normalizing to 5S small nuclear RNA using the 2^−ΔΔCt^ method (Ct refers to the threshold value).

Total RNA was isolated from HuREC using RNeasy kit (Qiagen, Germantown, MD, USA) according to the manufacturer’s protocol. cDNA was prepared using iScript^TM^cDNA Synthesis Kit (Bio-Rad, Hercules, CA, USA). Amplification of peroxisome proliferator-activated receptor gamma coactivator 1-alpha (PGC-1α), nuclear-encoded respiratory complex proteins (NRF-1) and mitochondrial transcription factor A (TFAM) were performed using Power SYBR green PCR master mix (Applied Biosystem, Foster City, CA, USA). The conditions used for the PCR were as it follows: 95 °C for 3 min (1 cycle) and 94 °C for 20 s, 55 °C for 30 s, and 72 °C for 40 s (40 cycles). Primer sequences used for qPCR are as follows. PGC-1α (forward): TCTGTGTCACTGTGGATTGGA, PGC-1α (reverse): AGTTCAGGAAGATCTGGGCAA, NRF-1 (forward): CGCGGTCGCAGTCTCC, NRF-1 (reverse): CGTGTTCCTCCATGAAGTTCTCA, TFAM (forward): ACCGAGGTGGTTTTCATCTGT and TFAM (reverse): TTTTGCATCTGGGTTCTGAGC. The relative mRNA abundance was determined by normalizing to 18s ribosomal RNA (rRNA) using the 2^−ΔΔCt^ method.

### 2.3. MiR-34a Over-Expression or Inhibition in HuREC

MiR-34a was over-expressed in HuREC by transfecting the cells with 50 or 100 nM of miScript hsa-miR-34a-5p mirVana^TM^ mimic or a scramble miR (Ambion, Waltham, MA, USA) used as negative control. Transfection of the cells was achieved by using Lipofectamine 2000 (ThermoFisher, Waltham, MA, USA) according to the manufacturer’s instruction. Similarly, HuREC were transfected with miScript has-miR-34a-5p mirVana^TM^ inhibitor (miR-34a IB; Ambion, Waltham, MA, USA) or miRNA inhibitor negative control (NC; Ambion, Waltham, MA, USA) and were exposed to the different glucose conditions (NG-, LG-, and HG-containing medium) as described previously.

### 2.4. Senescence-Associated β-Galactosidase Activity Assay

Senescence-associated β-galactosidase (SA-β-Gal) reactivity-based assay was performed to evidence senescent HuREC using a commercially available kit (Cell Signaling, Danver, MA). The cells were fixed with 2% formaldehyde and 0.2% glutaraldehyde in PBS. Positive reactivity to SA-β-Gal is assessed at pH 6 only in senescent cells, and is not found in pre-senescent, quiescent, or immortal cells. Images were captured at 20× magnification by light microscopy using Zeiss Axioplan2 (Carl Zeiss Microscopy, Thornwood, NY). Images were captured at 10 frames per well. Percentage of SA-β-Gal positive cells/well was determined as number of cells positive for a blue color versus total number of cells counted in a blind fashion. 

### 2.5. Protein Detection

Immunoblotting was performed to assess protein expression in response to the different treatments, as previously described [31]. Briefly, control and treated HuREC were lysed using radioimmunoprecipitation assay buffer lysis buffer containing 1% phosphatase and protease inhibitor cocktail (Sigma-Aldrich, St. Louis, MO, USA). Fifty micrograms of each protein sample was electrophoresed on SDS-PAGE and transferred onto a Polyvinylidene difluoride (PVDF) membrane. The membrane was then blocked using 5% skim milk and incubated with the following primary antibodies: SIRT1 (1:1000; Cell Signaling) and p16^INK4a^, TrxR2 (1:1000; Abcam, Cambridge, MA), p21^Waf1^ and SOD2 (1:500; Santa Cruz Biotech, Dallas, TX, USA), and corresponding secondary horseradish-conjugated antibodies (GE Healthcare, Pittsburg, PA, USA). After immunoblotting, the membranes were stripped using stripping buffer (Bio-Rad) and re-probed with anti-β-actin antibody (1:3000; Sigma-Aldrich, St. Louis, MO, USA). Chemiluminescence-based assay was used for band detection (ThermoFisher, Waltham, MA, USA). Scanned images of blots were used to quantify protein expression using NIH ImageJ software (http://rsb.info.nih.gov/ij/).

### 2.6. Assessment of Mitochondrial Function

Mitochondrial function was assessed using Seahorse extracellular flux (XFp) 96 analyzer [32] (Seahorse Bioscience, Inc, North Billerica, MA, USA), which measures the oxygen consumption rate (OCR), an indicator of mitochondrial respiration. Briefly, HuREC were plated at 10,000 cells/well and treated with HG only or transfected with miR-34a mimic for different time intervals. At the end of each treatment period, plates were loaded onto a Seahorse analyzer, and different pharmacological agents were sequentially added to respiring cells. For each parameter, three repeated rates of oxygen consumption were made over an 18 min period. Several measures of mitochondrial respiration, including basal respiration, ATP production, and spare respiratory capacity were derived using the recommended formulas. First, baseline cellular oxygen consumption was measured, from which basal respiration was derived by subtracting non-mitochondrial respiration. Next, oligomycin, an inhibitor of complex V, was added, and the resulting OCR was used to derive ATP-linked respiration (by subtracting the oligomycin rate from baseline cellular OCR). Next, carbonyl cyanide-p-trifluoromethoxyphenylhydrazone (FCCP), a protonophore, was added to collapse the inner membrane gradient, driving the ETC to function to its maximal rate, and maximal respiratory capacity was derived by subtracting non-mitochondrial respiration from the FCCP OCR. Lastly, antimycin A, a complex III inhibitor, and rotenone, a complex I inhibitor, were added to shut down ETC function, revealing the non-mitochondrial respiration. The mitochondrial reserve capacity was calculated by subtracting basal respiration from maximal respiratory capacity.

### 2.7. Reactive Oxygen Species Assays

Detection of reactive oxygen species (ROS) from cellular source was assessed by CellROX green assay (ThermoFisher, Waltham, MA, USA) according to the manufacturer’s protocol. HuREC were loaded with 5 µM CellROX green in culture medium and stained in the dark for 30 min at 37 °C. Stained cells were washed in PBS, mounted using Fluoroshield mounting medium containing 4′,6-diamidino-2-phenylindole (DAPI) to contemporaneously visualize nuclei. Images were then immediately captured using Zeiss Axioplan-2 Imaging florescence. 

Mitochondrial superoxide production was measured using MitoSOX red (ThermoFisher, Waltham, MA, USA), a fluorescent probe targeted to the mitochondria and specific for superoxide. HuREC were loaded with 5 µM MitoSOX red in Hank’s balanced salt solution (HBSS) with calcium and magnesium for 30 min at 37 °C in the dark. Stained cells were then washed and suspended in HBSS, mounted using Fluoroshield mounting medium containing DAPI and immediately analyzed under a Zeiss Axioplan-2 Imaging fluorescence.

### 2.8. Fluorescence-Activated Cell-Sorting (FACS) Analysis for Mitochondrial Membrane Potential and Density

To further determine mitochondrial membrane potential and density we monitored changes in florescence intensity of TMRE and MitoTracker dyes using FACS. At the end of the treatment period, HuREC cells were stained with 50 or 100 nM tetramethylrhodamine ethyl ester perchlorate (TMRE) MitoTracker for 30 min at 37 °C in the dark. After washing two times, the samples were analyzed using a flow cytometer (FACS caliber, BD Biosciences, San Jose, CA, USA).

### 2.9. Luciferase Assay

MiR-34a inhibitory activity towards specific RNAs was assessed by luciferase assay. HuREC were transfected with 100 nM miScript has-miR-34a-5p mirVana mimic (Ambion, Waltham, MA, USA) and co-transfected either with SIRT1 3`UTR target reporter clone, TrxR2 3`UTR target reporter clone, or SOD2 3`UTR target reporter clone (Gene Copoeia, Rockville, MD, USA). MiRNA target clone control vector and miRNA mimic negative control (Ambion, Waltham, MA, USA) were used as controls. Assays were performed using Secrete-Pair^TM^ dual luminescence assay kit (Gene Copoeia, Rockville, MD, USA). Briefly, cell culture medium was collected from the cells transfected with Gluc/SEAP dual reporter clone (Gene Copoeia, Rockville, MD, USA) for 48 h. For Gluc/SEAP assay, 100 µL of luminescent and 10 µL of the collected supernatant was used per the reaction in a 96-well plate (in triplicate); the readings were taken using a luminometer (Molecular Devices Gemini XS Fluorescent Microplate Reader; Marshal Scientific, NH, USA). The luciferase units were measured as relative luciferase units (RLU) and normalized to total protein.

### 2.10. Statistical Analysis

The results are presented as mean ± S.E.M for a minimum of four independent experiments. Statistical significance was set as *p* < 0.05 and determined using one way ANOVA and Student’s t-test using GraphPad Prism 7 software (GraphPad, San Diego, CA, USA).

## 3. Result

### 3.1. High Glucose Stimulates miR-34a Expression and Promotes HuREC Senescence 

**Figure 1:**
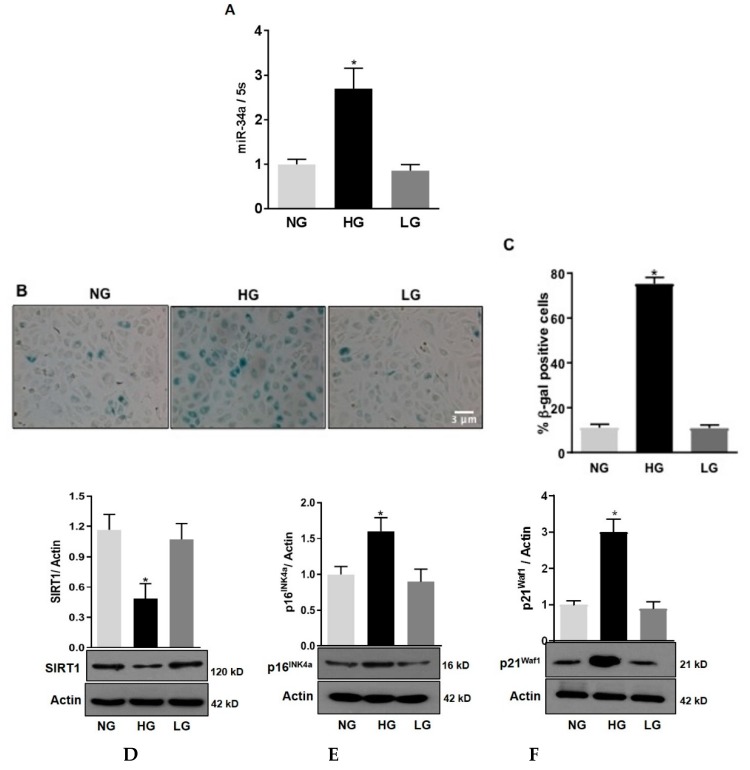
Effects of high glucose on miR-34a and endothelial cells senescence. (**A**) Bar histograms representing miR-34a levels measured by qPCR in HuREC treated for 48 h with high glucose (HG; 25 mM d-glucose, black bar) in comparison to cells treated with normal glucose (NG; 5 mM D-glucose, light grey bar) or the osmotic control l-glucose (LG; 25 mM L-glucose, dark grey bar). (**B**) Micro images showing SA-β-Gal (senescence-associated beta-galactosidase) staining (blue cells) in HuREC exposed for 72 h to different glucose concentrations (NG and HG) or the osmotic control LG. (**C**) Bar histograms representing percentage of SA-β-Gal positive cells versus total number of cells counted per field. Ten consecutive fields per sample were counted in a blinded fashion. (**D**–**F**) Immunoblotting analysis determining protein levels of (**D**) SIRT1, (**E**) p16INK4a, and (**F**) p21Waf1 in HuREC exposed to HG for 72 h (black bar) and compared to NG (light grey bar) and LG (dark grey bar) controls. Protein levels are expressed (bar histograms) as band density normalized versus actin. Bars represents mean value ± S.E.M; * *p* < 0.05 vs. NG, *n* = 4.

We have previously shown that, in retinas of diabetic rats, the expression of miR-34a is increased [3]. Here we aimed at evaluating the role of miR-34a specifically in retinal endothelial cells. QPCR analysis showed that in HuREC exposed to HG conditions (25 mM d-glucose) there was a significant increase in the expression levels of miR-34a at 48 h (*p* < 0.01 vs. NG; Figure 1A). This effect was not observed when HuREC were cultured in presence of the osmotic control. Parallel to miR-34a expression, SA-β-Gal activity was significantly augmented in cells exposed to HG (*p* < 0.001; Figure 1B,C) when compared to controls (NG and LG). Immunoblotting showed that HG significantly inhibited the expression of the histone deacetylase SIRT1 (*p* < 0.01; Figure 1D) and increased protein levels of the senescence markers p16^Ink4a^ (*p* < 0.01; Figure 1E) and p21^Waf1^ (*p* < 0.001; Figure 1F) when compared to cells treated with NG and LG. These data confirm that glucidic stress stimulates miR-34a and promotes HuREC senescence.

### 3.2. High Glucose Promotes Mitochondrial ROS Production in HuREC 

**Figure 2:**
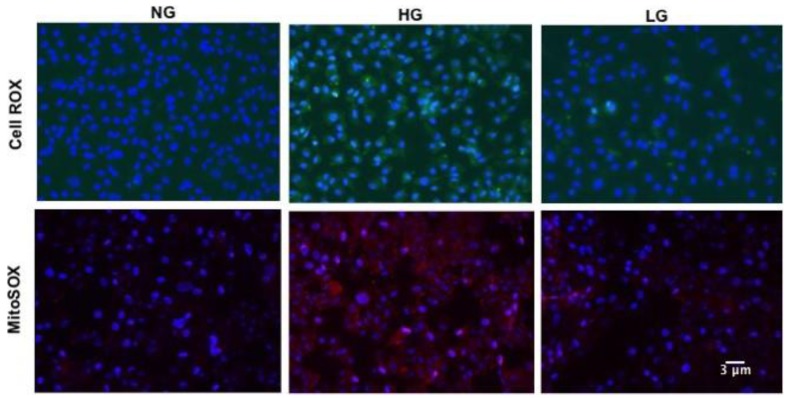
Effects of high glucose on cellular and mitochondrial reactive oxygen species (ROS). Upper panels: Micro images of CellROX-stained cells showing cellular ROS formation (green florescence) in HuREC treated for 48 h with high glucose (HG; 25 mM d-glucose), normal glucose (NG; 5 mM d-glucose), and the osmotic control L-glucose (LG; 25 mM l-glucose). Lower panels: Micro images of MitoSOX stained cells showing mitochondrial ROS formation (red florescence) in HuREC treated for 48 h with high glucose (HG; 25 mM d-glucose), normal glucose (NG; 5 mM d-glucose), and the osmotic control L-glucose (LG; 25 mM l-glucose). DAPI was used for nuclear counterstain (blue fluorescence).

Retinal vascular senescence is associated with increased oxidative stress [3]; therefore, we measured ROS production in HuREC exposed to HG (Figure 2, upper panels). CellROX-based analysis showed that HG stimulated cellular ROS production as compared to NG or LG controls (Figure 2, upper panels).

Since loss of SIRT1 and vascular senescence are linked to mitochondrial dysfunction [33], we also assessed mitochondrial ROS production using MitoSOX mitochondrial superoxide indicator. As shown in Figure 2 (lower panels), enhanced fluorescence signaling was observed in HG-stimulated HuREC when compared to NG and LG controls, thus indicating increased mitochondrial ROS production. 

**Figure 3:**
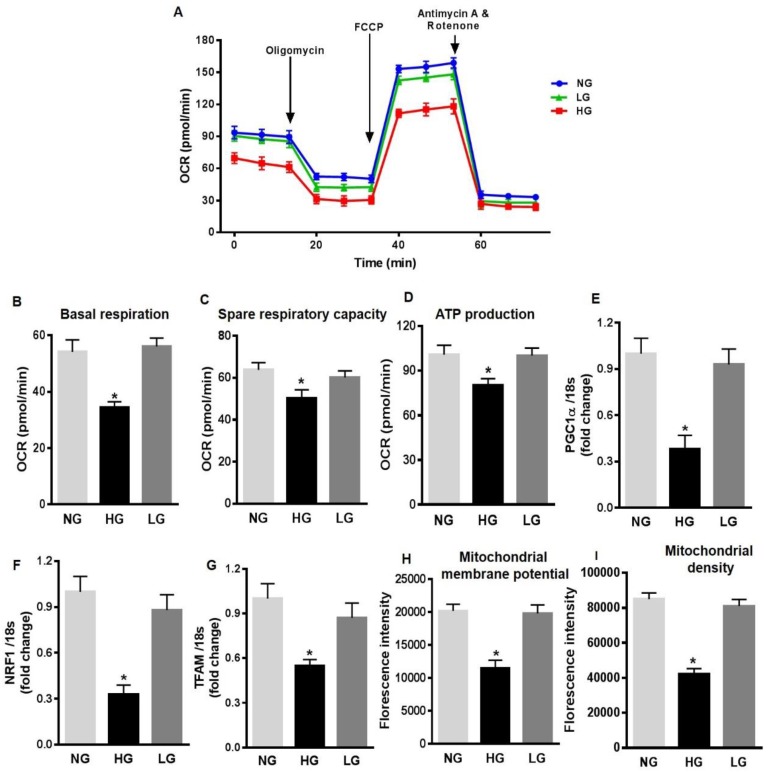
High glucose affects mitochondrial respiration and biogenesis in HuREC. (**A**) Oxygen consumption rate (OCR) trace was determined using a Seahorse XFp analyzer in HuREC treated for 48 h with HG (25 mM d-glucose, black bar) in comparison to cells treated with NG (5 mM d-glucose, light grey bar), or the osmotic control LG (25 mM l-glucose, dark grey bar). (**B**) Baseline, (**C**) spare respiratory capacity, and (**D**) ATP production were all decreased in HuREC treated with HG compared to NG and LG controls. (**E**–**F**) qPCR analysis assessing mRNA levels of genes regulating mitochondrial biogenesis: Peroxisome proliferator-activated receptor gamma coactivator 1-alpha (PGC-1α), nuclear-encoded respiratory complex proteins (NRF-1), and mitochondrial transcription factor A (TFAM). Bar histogram representing mRNA levels of (**E**) PGC-1α, (**F**) NRF-1, and (**G**) TFAM genes in HuREC treated for 48 h with HG (black bar), NG (light grey bar), and LG (dark grey bar). In all cases (**D**–**F**) data are normalized to 18s expression and represented as fold of change. FACS analysis of (**H**) TMRE and (**I**) MitoTracker dyes of HuREC treated for 48 h with HG (black bar), NG (light grey bar), and LG (dark grey bar). Bars represent mean value ± S.E.M; * *p* < 0.05 *vs*. NG, *n* = 4.

To further investigate the mechanisms of increased mitochondrial ROS production in HG-stimulated HuREC, we measured different mitochondrial respiration parameters. HuREC stimulated with HG were sequentially treated with oligomycin (complex V inhibitor), FCCP (mitochondrial membrane depolarization), rotenone (complex I inhibitor), and antimycin (Ant A-complex III inhibitor). HG-treated cells showed significant lower basal respiration (Figure 3A,B; *p* < 0.001) than the controls, NG and LG, indicating an alteration in ATP turnover. Spare respiratory capacity reflects the ability of the cells to respond to an increase in ATP demand. We observed a significantly reduced spare respiratory capacity (*p* < 0.001; Figure 3C) and rate of ATP production (*p* < 0.01; Figure 3D) in HG-treated cells when compared to the NG and LG. These data indicate increased mitochondrial stress in HG-stimulated cells.

Next, we measured the expression of genes regulating mitochondrial biogenesis, such as PGC-1α, NRF-1, and TFAM. QPCR analysis showed that in HuREC, HG significantly decreased expression of PGC-1α (*p* < 0.01; Figure 3E), NRF-1 (*p* < 0.001; Figure 3F), and TFAM (*p* < 0.01, Figure 3G) when compared to controls. These data indicate that HG alters mitochondrial function and biogenesis in HuREC. Finally, to further confirm mitochondrial dysfunction, we evaluated changes in mitochondrial membrane potential and in density of healthy respiring mitochondria using TMRE and MitoTracker dyes, respectively, by FACS analysis. As shown in Figure 3H–I, HG exposure to HuREC significantly reduced mitochondrial membrane potential and density of healthy respiring mitochondria, as indicated by lower florescence intensity with TMRE and MitoTracker dyes.

### 3.3. MiR-34a Inhibition Prevents High Glucose-Induced Vascular Senescence and Oxidative Stress in HuREC

**Figure 4:**
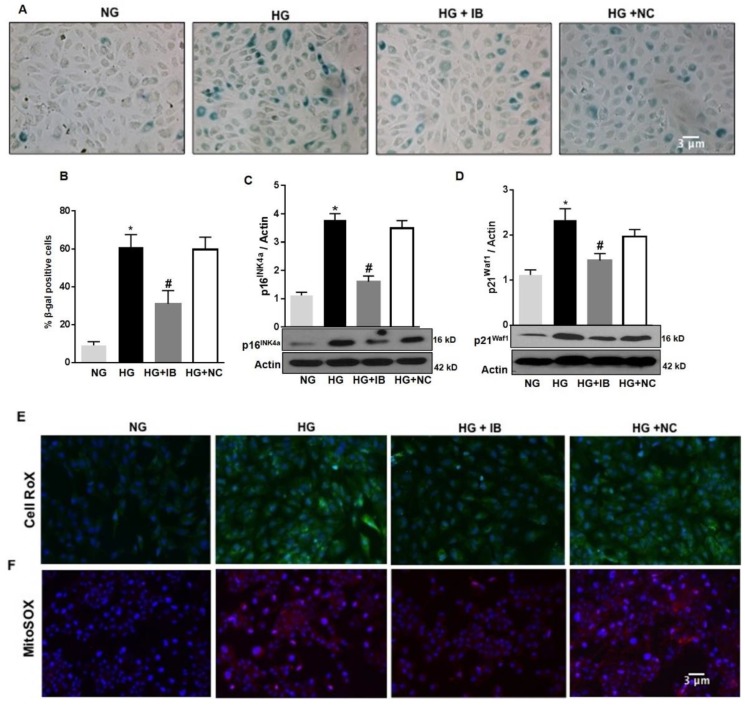
MiR-34a inhibition prevents high glucose-induced vascular senescence and oxidative stress in HuREC. HuREC were transfected with 50 nM of miR-34a inhibitor (miR-34a IB) or negative control (NC) for 24 h and exposed to HG (25 mM d-glucose) or NG (5 mM d-glucose) or the osmotic control LG (25 mM l-glucose) for 72 h. (**A**) Micro images showing SA-β-Gal staining (blue cells) in non-transfected HuREC treated with NG or HG for 72 h or HuREC transfected with miR-34a IB or negative control. (**B**) Bar histograms representing percentage of SA-β-Gal positive cells versus total number of cells counted per field from different experimental groups as described above. Ten consecutive fields per sample were counted in a blinded fashion. (**C**,**D**) Immunoblotting analysis determining protein levels of (**C**) p16^INK4a^ and (**D**) p21^Waf1^ in HuREC treated with NG (light gray bar) or HG (black bar) for 48 h without transfection, or HG transfected with miR-34a IB (dark gray bar) or negative control (white bar). Changes in protein levels are expressed (bar histograms) as band density normalized versus actin. Micro images showing (**E**) cellular ROS production using CellROX (green florescence) and (**F**) mitochondrial superoxide production using MitoSOX stained (red florescence) in HuREC transfected with miR-34a IB or NC and exposed to HG for 48 h. DAPI (blue florescence) was used for nuclear counterstains. Bars represent mean value ± S.E.M; * *p* < 0.05 *vs*. NG and #*p* < 0.05 *vs*. HG, *n* = 4.

To determine whether up-regulation of miR-34a contributes to HG-induced vascular senescence and increased ROS production, we examined the effects of inhibiting miR-34a activity by transfecting the cells with a specific antagomiR (miR-34a inhibitor = IB). As previously shown, HG increased the number of SA-β-Gal activity when compared to control (NG). Transfection of these cells with miR-34a inhibitor significantly reduced the number of SA-β-Gal positive cells (*p* < 0.01; Figure 4A,B), although their number remained higher than control (NG). This effect was not seen in HuREC transfected with a non-specific antagomiR (NC). Immunoblotting showed that transfection of the cells with miR-34a inhibitor, but not the miR control, significantly diminished HG-induced protein levels of the senescence markers p16^Ink4a^ (*p* < 0.01; Figure 4C) and p21^Waf1^ (*p* < 0.01; Figure 4D). 

Further, transfection of HuREC with miR-34a inhibitor significantly diminished the number of cells reactive to CellROX (Figure 4E) and MitoSOX (Figure 4F) in response to HG stimulation, suggesting that miR-34a inhibitor reduced cellular and, especially, mitochondrial ROS production elicited by glucidic stress. 

**Figure 5:**
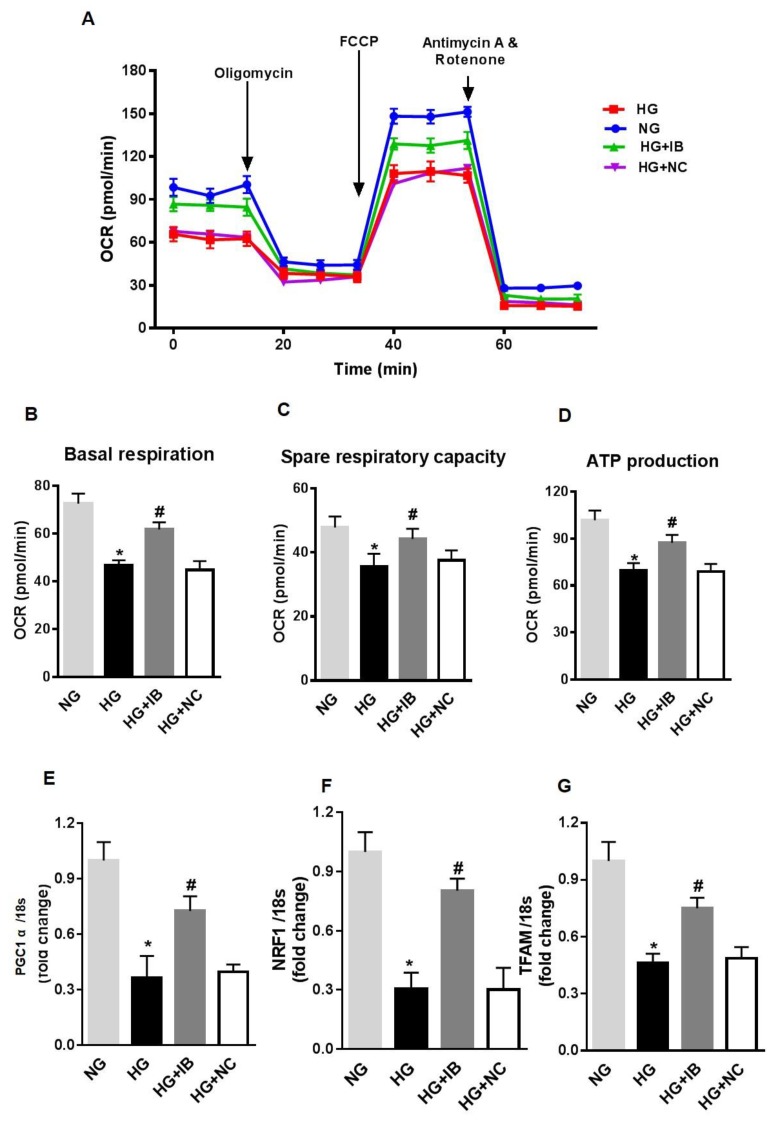
MiR-34a inhibition rescues high glucose-induced changes in mitochondrial respiration and biogenesis in HuREC. Mitochondrial function was assessed using the Seahorse XFp analyzer in HuREC transfected with 50 nM of miR-34a inhibitor (IB) or negative control (NC) for 24 h and exposed to high glucose (HG; 25 mM d-glucose) for 48 h. (**A**) oxygen consumption rate (OCR), (**B**), baseline, (**C**) spare respiratory capacity, and (**D**) ATP production are shown. (**E**–**G**) qPCR analysis assessing mRNA levels of genes regulating mitochondrial biogenesis: PGC-1α, NRF-1, and TFAM. Bar histogram representing mRNA levels of (**E**) NRF-1, (**F**) PGC-1α, and (**G**) TFAM genes in HuREC treated with NG (light gray bar) or HG (black bar) following without transfection or transfected with miR-34a IB (dark gray bar) and negative control (white bar). Bars represent mean value ± S.E.M; * *p* < 0.05 *vs*. NG and #*p* < 0.05 *vs*. HG, *n* = 4.

Finally, to determine the effects of miR-34a on HG-induced mitochondrial dysfunction, we assessed the effects of miR-34a inhibition on mitochondrial function and expression of mitochondrial biogenesis genes. HuREC were transfected with miR-34a inhibitor or scramble control and, subsequently, exposed to HG or to NG. After 48 h, the cells were assessed for mitochondrial function measuring OCR (Figure 5A–D) and expression of biogenesis genes. Inhibition of miR-34a rescued spare respiratory capacity (*p* < 0.01; Figure 5C) and ATP production (*p* < 0.001; Figure 5D). Furthermore, inhibition of miR-34a significantly blunted the HG-induced decrease in mRNA expression levels of PGC-1α (*p* < 0.01; Figure 5E), NRF-1 (*p* < 0.01, Figure 5F), and TFAM (*p* < 0.01; Figure 5G).

These results indicate that HG-mediated up-regulation of miR-34a in HuREC is directly associated with mitochondrial dysfunction and consequent elevation of ROS production and senescence markers.

### 3.4. MiR-34a Mimic Triggers Vascular Senescence and ROS Production in HuREC 

**Figure 6:**
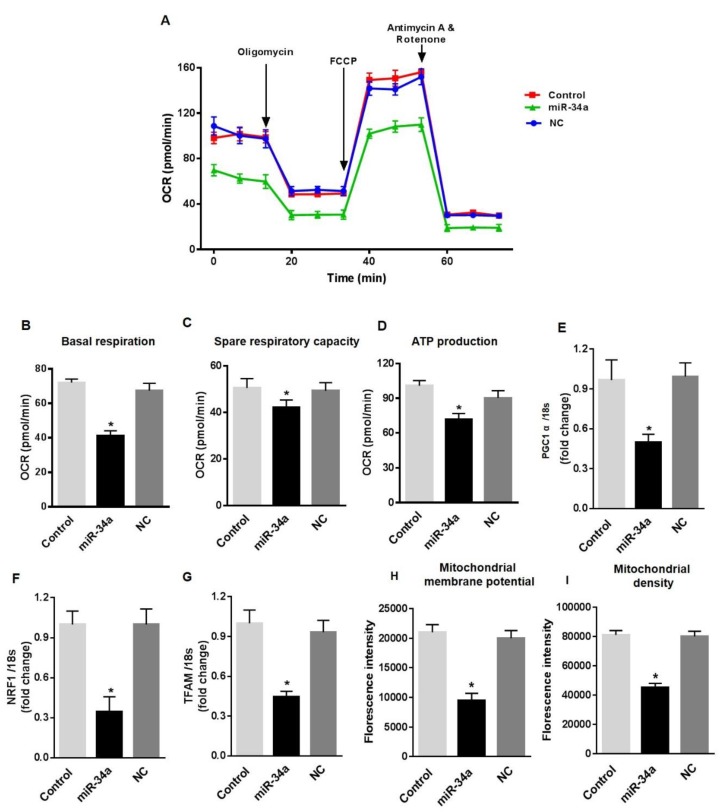
MiR-34a overexpression affects mitochondrial respiration and biogenesis, and induces senescence in HuREC. Mitochondrial function was assessed using the Seahorse XFp analyzer in HuREC transfected with 50 nM of miR-34a mimic (miR-34a) or negative control (NC) for 48 h. Non-transfected HuREC cells were used as control. Bar graphs of (**A**) OCR, (**B**) baseline, (**C**) spare respiratory capacity, and (**D**) ATP production in HuREC without transfection (light gray) or transfected with miR-34a mimic (black bar) or negative control (dark gray bar) for 48 h. (**E**–**G**) qPCR analysis assessing mRNA levels of genes regulating mitochondrial biogenesis: PGC-1α, NRF-1, and TFAM. Bar histogram representing mRNA levels of (**E**) PGC-1α, (**F**) NRF-1, and (**G**) TFAM genes in HuREC without transfection (light gray) or transfected with miR-34a mimic (black bar) or negative control (dark gray bar) for 48 h. FACS analysis assessing TMRE (**H**) and MitoTracker (**I**) dyes signals in HuREC without transfection (light gray) or 48 h after transfection with miR-34a mimic (black bar) or with negative control (dark gray bar). Bars represent mean value ± S.E.M; * *p* < 0.05 *vs*. Control or NC, *n* = 4.

To further confirm the dysfunctional effects of miR-34a elevation in retinal endothelial cells, we assessed the consequences of the direct overexpression of miR-34a in HuREC cultured in NG. HuREC were transfected with 50 nM of miR-34a mimic or with 50 nM of scramble miR, used as control for off-target effects. Forty-eight hours post-transfection, the cells were analyzed for mitochondrial function and senescence-like markers. Transfection of the cells with miR-34a mimic promoted a significant depression of basal respiratory capacity (*p* < 0.01; Figure 6A,B), spare respiratory capacity (*p* < 0.01; Figure 6C), and rate of ATP production (*p* < 0.01; Figure 6D). Overexpression of miR-34a in HuREC also significantly reduced the expression levels of PGC-1α (*p* < 0.01; Figure 6E), NRF-1(*p* < 0.01; Figure 6F) and TFAM genes (*p* < 0.01; Figure 6G), and reduced mitochondrial membrane potential (Figure 6H) and density of healthy mitochondria (Figure 6I), thus further confirming the role of miR-34a in suppressing mitochondrial function and biogenesis for all the different parameters analyzed. The scramble control (NC) had no effects, thus confirming the specificity of the results observed by transfection of HuREC with the miR-34a mimic.

**Figure 7:**
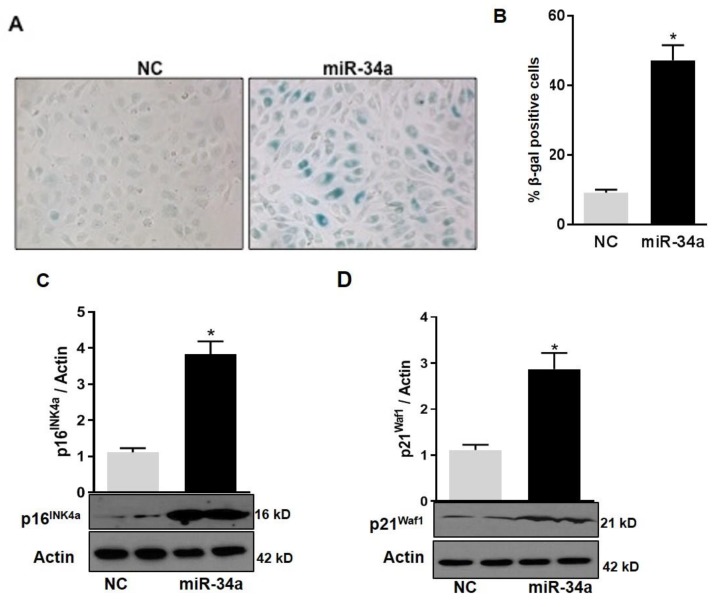
MiR-34a overexpression induces senescence in HuREC. (**A**) Micro images showing SA-β-Gal staining (blue cells) in HuREC transfected with miR-34a mimic or negative control for 48 h. (**B**) Bar histograms representing percentage of SA-β-Gal positive HuREC (per field) transfected with miR-34a mimic (black bar) or negative control (dark gray bar) for 48 h. Ten consecutive fields per sample were counted in a blinded fashion. Immunoblotting analysis determining protein levels of (**C**) p16^INK4a^ and (**D**) p21^Waf1^ in HuREC transfected with miR-34a mimic (black bar) or negative control (dark gray bar) for 48 h. Changes in protein levels are expressed (bar histograms) as band density normalized versus actin. Bars represent mean value ± S.E.M; * *p* < 0.05 *vs*. NC, *n* = 4.

In agreement with the notion that mitochondrial dysfunction in HuREC leads to accelerated cell senescence, we also found that overexpression of the miR-34a mimic in HuREC increased SA-β-Gal activity (Figure 7A,B) and the levels of the senescence markers p16^Ink4a^ (*p* < 0.001; Figure 7C) and p21^Waf1^ (*p* < 0.001; Figure 7D), measured by immunoblotting, as compared to cells transfected with scrambled miR (NC).

### 3.5. Inhibition of MiR-34a Halts High Glucose-Induced Loss of SIRT1, SOD2, and TrxR2

**Figure 8:**
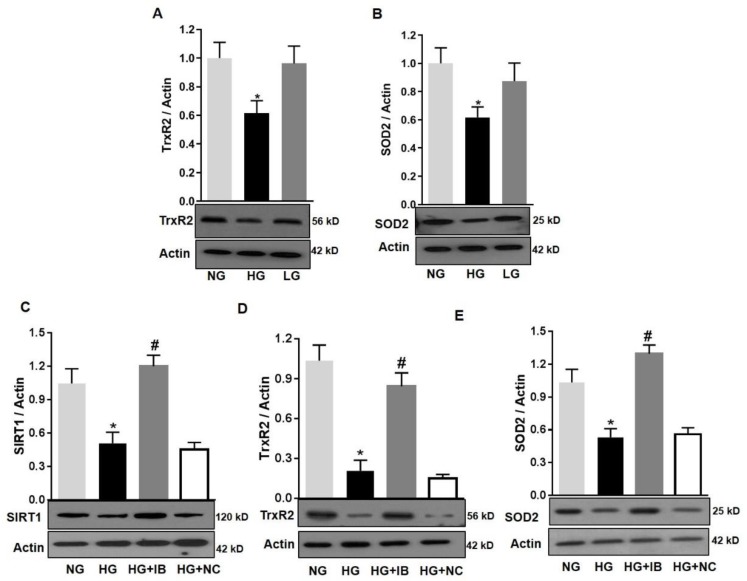
MiR-34a inhibition improves mitochondrial antioxidants in high-glucose-exposed HuREC. HuREC were treated for 48 h with high glucose (HG; 25 mM d-glucose, black bar) or normal glucose (NG; 5 mM d-glucose, light grey bar) or the osmotic control l-glucose (LG; 25 mM l-glucose, dark grey bar). Immunoblotting analysis showing the protein levels of (**A**) TrxR2 and (**B**) SOD2 in HuREC exposed to HG (black bar) and compared to NG (light grey bar) and LG (dark grey bar) controls. (**C**–**E**) HuREC were transfected with 50 nM of miR-34a inhibitor (miR-34a IB) or negative control (NC) for 24 h and exposed for 48 h with HG, NG, or LG. Immunoblotting analysis determining protein levels of (**C**) SIRT1, (**D**) TrxR2, and (**E**) SOD2 in HuREC treated with NG (light gray bar) or HG (black bar) alone or followed by transfection with miR-34a IB (dark gray bar) and negative control (white bar). Changes in protein levels are expressed (bar histograms) as band density normalized versus actin. Bars represent mean value ± S.E.M; * *p* < 0.05 vs. NG and # *p* < 0.05 *vs*. HG, *n* = 4.

The effects of miR-34a on mitochondrial function of retinal microvascular endothelial cells are likely to be due to downregulation of SIRT1, its validated target [12,13]. However, other potential targets of miR-34a include key mitochondrial endogenous antioxidant such as TrxR2 [30]. Moreover, SOD2 is a key mitochondrial antioxidant [34]. We performed immunoblotting analysis to assess the expression of these antioxidants in HuREC in response to HG treatment. This analysis determined that HG (48 h) significantly reduced protein levels of TrxR2 (*p* < 0.01; Figure 8A) and SOD2 (*p* < 0.01; Figure 8B) in comparison to NG and the osmotic control LG. Furthermore, loss of SIRT1, TrxR2, and SOD2 protein levels in response to HG was significantly blocked by transfection of the cells with the miR-34a inhibitor (*p* < 0.01 for all; Figure 8C–E, respectively), but not with the scramble antagomiR (NC). Therefore, these data support the direct cause–effect relationship between up-regulation of miR-34a in HG and loss of mitochondrial endogenous antioxidants. 

### 3.6. MiR34a Mimic Inhibits the Expression of SOD2, TrxR2, and SIRT1

**Figure 9:**
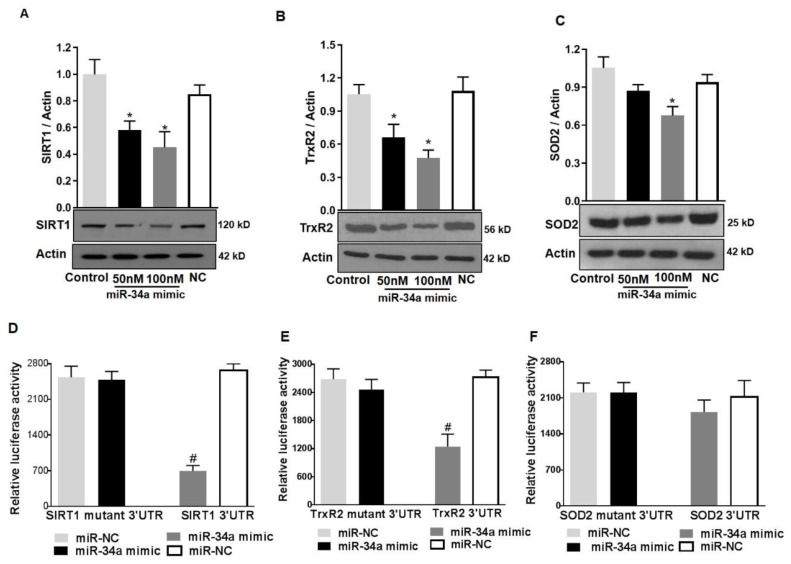
MiR-34a targets SIRT1 and TrxR2 but not SOD2 in HuREC. HuREC were transfected with 50 or 100 nM of miR-34a mimic or negative control (NC). Non-transfected HuREC cells were used as control. Immunoblotting analysis determining protein levels of (**A**) SIRT1, (**B**) TrxR2, and (**C**) SOD2 in HuREC transfected with 100 nM miR-34a mimic (black and dark gray bars), negative control (white bar), or control (light gray bar). Results of luciferase assay obtained to determine miR-34a binding to 3’-UTR binding of (**D**) SIRT1, (**E**) TrxR2, or (**F**) SOD2. HuREC were transfected with plasmid containing 3’-UTR of target gene (SIRT1, TrxR2, or SOD2) or plasmid containing mutated 3′-UTR of target gene alone or in combination with miR-34a mimic. All transfections were carried out for 48 h and relative luciferase activity were determined using a commercially available luciferase assay kit. Bars represent mean value ± S.E.M; * *p* < 0.05 *vs*. control and # *p* < 0.05 *vs*. miR-NC, *n* = 4.

To further confirm the direct contribution of miR-34a in modulating SOD2 and TrxR2 protein levels, we determined the effects of overexpressing a miR-34a mimic on the expression of mitochondrial antioxidants in HuREC cultured in normal glucose conditions (NG). Cells were transfected with 50 and 100 nM of miR-34a mimic and compared with cells transfected with a scramble control (100 nM, NC). After 48 h, the cells were lysed, protein extracted, and immunoblotting was performed to determine SIRT1 (used as positive control), TrxR2, and SOD2 protein levels. As shown in Figure 9, transfection of the cells with miR-34a mimic significantly decreased protein levels of SIRT1 (*p* < 0.01; Figure 9A) and TrxR2 (*p* < 0.01; Figure 9B) and, to a lesser extent, although statistically significant, SOD2 (*p* < 0.01; Figure 9C). 

### 3.7. SIRT1 and TrxR2 but Not SOD2 Are Direct Targets of MiR34a

As previously indicated, while SIRT1 is a validated target of miR-34a [12,13], TrxR2 is the predicted one [30]. Since there was a concomitant decrease in SOD2 expression along with TrxR2, we also determined if miR-34a would directly target SOD2. We; therefore, investigated miR-34a direct inhibitory activity towards TrxR2 and SOD2 RNAs, using a luciferase reporter assay. HuREC were co-transfected with 100 nM mimic and TrxR2 3’UTR or SOD2 3’UTR target reporter clones. MiR-34a inhibitory activity was also tested on SIRT1 3’UTR target reporter clone as a positive control for the assay. Transfection of the cells with mutant 3’UTR for each specific RNA was also performed to control for specificity of the reaction. Finally, as control for off-target side effects, cells were co-transfected with a scramble miR (NC) and each reporter clone. MiR-34a mimic significantly inhibited luciferase activity from TrxR2 3’UTR (*p* < 0.01; Figure 9E) reporter and, as expected, from SIRT1 3’UTR reporter (*p* < 0.01; Figure 9D), but not from SOD2 3’UTR reporter (Figure 9F). This effect was specific as co-transfection of the cells with the scramble mimic had no effect and transfection of the cells with mutants 3’ UTR prevented miR-34a inhibitory activity. Overall, these data confirmed that, in HuREC, SIRT1 and TrxR2 are direct targets of miR-34a, whereas SOD2 is not.

## 4. Discussion

Diabetic retinopathy is characterized by the interdependent succession of pathogenic events affecting both vascular and neuroretina [1,2]. Recent advances in our understanding of retinal microvascular dysfunction in diabetes showed the occurrence of stress-associated premature senescence of the retinal microvasculature in the diabetic milieu [35].

To unravel the molecular mechanisms explaining vascular endothelial cell senescence in the diabetic retina, we have conducted studies aimed at elucidating the specific contribution of miR-34a to this mechanism in human retinal microvascular endothelial cells.

The contribution of miR-34a to vascular senescence and apoptosis has been previously documented [12,14,15,18], and increased expression of this miR has been found in human and experimental diabetes [22,23,24]. Our group and others have previously shown that miR-34a levels are increased in retinas of diabetic rats [3,36] and this effect is associated with retinal vascular senescence and loss of expression and activity of SIRT1, a validated target of this miR [12,37].

In agreement with our previous findings in animal models of DR, our present results show that exposure of HuREC to disease-relevant doses of glucose (HG) resulted in increased miR-34a expression and acquisition of senescence-associated features accompanied by loss of SIRT1. Most importantly, inhibition of miR-34a, achieved by transfection of the cells with a specific antagomiR (IB), prevented HG effects on cell senescence and rescued SIRT1 expression. On the other hand, overexpression of miR-34a mimic in HuREC cultured in NG recapitulated the effects of HG by up-regulating senescence markers and inhibiting SIRT1 expression, thus further confirming the direct involvement of miR-34a in the observed effects.

Due to the known regulatory activity of SIRT1 on mitochondrial function and biogenesis [38,39], these results suggest a role for miR-34a in mediating HG-induced mitochondrial dysfunction. Consistent with this hypothesis, we found that antagomiR-34a (IB) rescued HG-induced ROS production from mitochondria (MitoSOX), while improving mitochondrial respiration and halting the loss of PGC-1α, NRF1, and TFAM, thus preserving mitochondrial biogenesis. Moreover, overexpression of miR-34a in HuREC elicited the same responses of HG by promoting mitochondrial dysfunction and ROS production and by halting mitochondria biogenesis due to loss of PGC-1α, NRF1, and TFAM

PGC-1α is a major regulator of mitochondrial biogenesis and has been shown to protect endothelial cells from ROS-induced dysfunction [40]. PGC-1α is also a transcriptional regulator of NRF1 and TFAM, which are involved in mitochondrial DNA replication and maintenance of mitochondria number [41,42]. Importantly, PGC-1α is required for SIRT1-dependent regulation of a set of genes involved in mitochondrial ROS detoxification [43]. Here, we showed that HG significantly blunted the expression of the mitochondrial antioxidants TrxR2 and SOD2, and blockade of miR-34a, by transfection of the cells with the specific anatgomiR-34a, rescued their expression. On the contrary, overexpression of the miR-34a mimic blunted the expression levels of these mitochondrial antioxidants, even in normal glucose conditions. These data, further confirmed the ability of miR-34a to directly promote retinal endothelial cells mitochondrial dysfunction.

The effects of miR-34a in suppressing TrxR2 and SOD2 expression could result as consequence of miR-34a-mediated loss of the SIRT1–PGC-1α signaling axis. However, previous studies conducted in aging renal cells [30] suggested that TrxR2 is a potential direct target of miR-34a.

The results of our studies examining the effects of miR-34a on the reporter 3’-UTR assays, analyzing both genes, confirmed that, at least in our experimental conditions, TrxR2 is a direct target of miR-34a, whereas SOD2 is not, thus suggesting that loss of SOD2 could be secondary to miR-34a-mediated loss of SIRT1, another important mechanism of miR-34a requiring further investigation.

## 5. Conclusions

**Figure 10:**
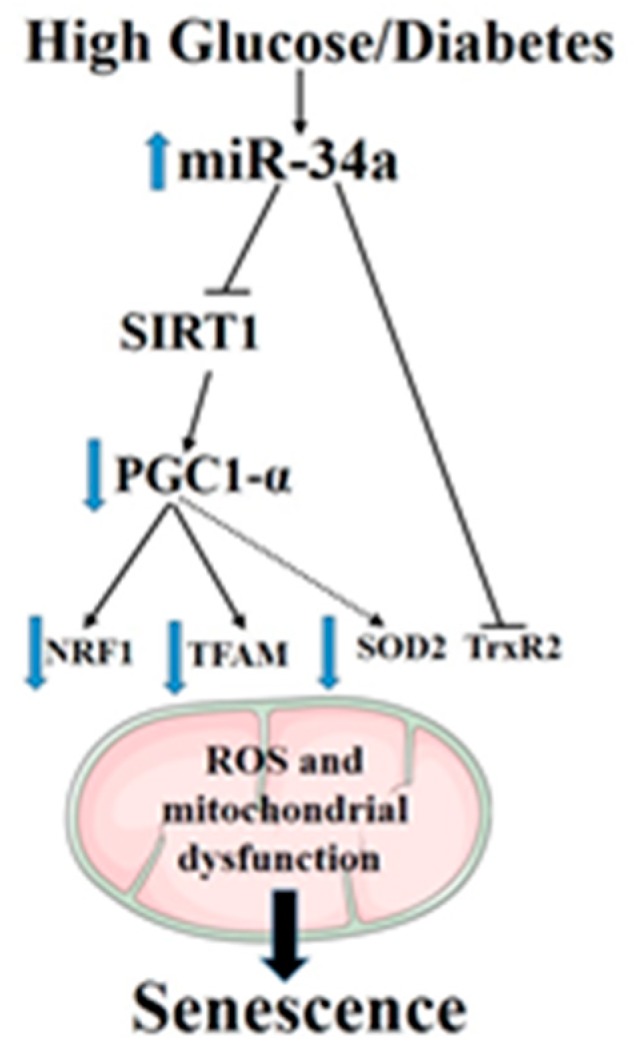
A summary diagram showing the role of miR-34a in regulating high-glucose-induced senescence in retinal endothelial cells.

In summary, the obtained data, by confirming the pathogenic role of miR-34a as a direct mediator of stress-associated premature senescence in retinal endothelial cells in response to glucidic stress, disclose the mechanism of action of this miR in altering mitochondrial function and impairing mitochondrial biogenesis by multiple pathways (Figure 10). These include suppression of the SIRT1–PGC-1α axis as well as the mitochondrial antioxidants TrxR2 and SOD2. Furthermore, we have validated TrxR2 as a direct target of miR-34a in HuREC, but we could not confirm SOD2. Nevertheless, the ability of miR-34a to affect the expression levels of SOD2 by indirect mechanisms is of particular interest, as recent evidence suggests that epigenetic silencing of this enzyme is implicated in diabetic memory [44,45], a key pathogenic mechanism for the development of DR and of other complications of diabetes. Further studies should be conducted to ascertain the direct involvement of miR-34a in this important and clinically-relevant mechanism and to determine the role of miR-34a as a predictor of DR progression.
